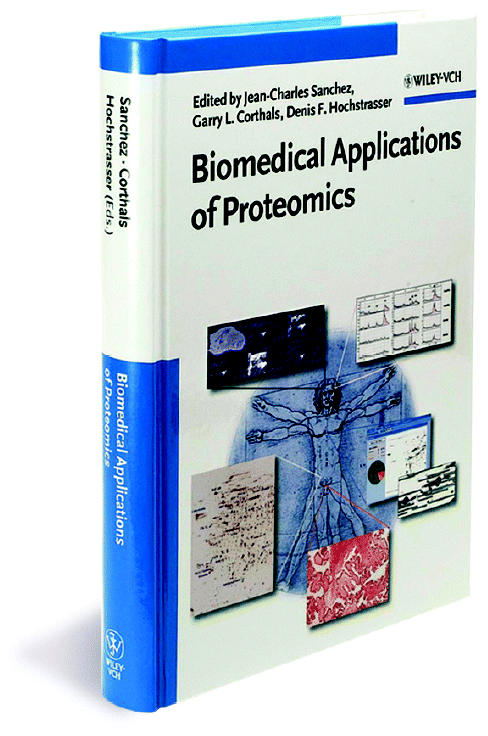# Biomedical Applications of Proteomics

**Published:** 2005-02

**Authors:** David R. Goodlett

**Affiliations:** David R. Goodlett is an associate professor in the Medicinal Chemistry Department at the University of Washington, where he directs mass spectrometry cores for bio-defense research in gram-negative bacteria and ecogenetics and environmental health. His research group is developing new informatics and mass spectrometric platforms for discovery-based proteomics research.

Edited by Jean-Charles Sanchez, Garry L. Corthals, and Denis F. Hochstrasser

Weinheim:Wiley-VCH, 2004. 435 pp. ISBN: 3-527-30807-5, $186 cloth

This book is aimed clearly at those wishing to understand what current proteomics technology can do for biomedical research. It is a compendium of biomedical problems analyzed using a wide array of state-of-the-art proteomic techniques. Those interested in quick tutorials of what proteomics can do from the perspective of various diseases or the organisms that cause them—such as stroke; vascular disease; cancers of the kidney, ovary, and colon; diabetes; human immunodeficiency virus; herpes simplex virus; *Francisella tularensis*; and central nervous system disorders—will find the book a great resource. Specifically, those new to the field can use the book to quickly catch up on applications of proteomics in these fields. Those outside these areas will also find the book a great resource for literature and techniques.

The book begins with a well-composed contemplation of the current and future value of proteomics to the clinical sciences by Marc Reymond, which includes a succinct discussion of the ethical implications of such research. The book is then segregated thematically. Five sections address biologic problems—blood vessels, cancer, pharmaco-toxicology, infectious disease, and the central nervous system. Each has several chapters written by experts who review the literature and discuss current advantages of proteomic research. The final section, on mass spectrometry and bioinformatics, appears to be incomplete, but one can easily argue that the pertinent information on most technical aspects of proteomics is already covered under the biologic sections. This final section reviews matrix-assisted laser desorption ionization mass spectrometry (MALDI-MS) imaging, a state-of-the-art technique that may eventually be used to aid traditional histologic interpretation of disease state. Also included is a chapter on the availability and use of proteomic databases. Though perhaps a bit bland, this topic is of utmost importance to understanding the implications of how proteins are identified and how they are annotated. Otherwise, in the current proteomics—where proteins are identified primarily by correlation of mass spectrometric data to sequence in a database, rather than *de novo* as during the age of chemical sequencing by Edman chemistry—there is great leeway for misinterpretation of proteomic data. The authors do a nice job of reviewing the informatics field at a high level, indicating where one may go for further information on any specific topic.

Each chapter describes variations on the implementation of currently available proteomics technology from the perspective of the biomedical scientists trying to better understand human disease. For the most part, the authors have used two-dimensional (2D) gel electrophoresis to solve their problems rather than the non-gel-based “shotgun” proteomic techniques. The book provides nice examples as justification for continued use of this original proteomic tool, but it is not exclusively about proteomics from the perspective of 1D or 2D gels. At some point just about all currently available proteomic discovery-based tools are discussed, and although the book is not organized by technique, one can locate discussion of pertinent techniques in the index.

Editors Corthals, Hochstrasser, and Sanchez are themselves expert innovators in the use of proteomics to better understand biology and medicine, and they have compiled a very nice resource written from the perspective of the biomedical practitioner of proteomics. Each chapter provides the novice in proteomics with insight into how these new discovery tools can be incorporated into traditional hypothesis-driven research.

## Figures and Tables

**Figure:**